# Ketamine Protects Gamma Oscillations by Inhibiting Hippocampal LTD

**DOI:** 10.1371/journal.pone.0159192

**Published:** 2016-07-28

**Authors:** Lanting Huang, Xiu-Juan Yang, Ying Huang, Eve Y. Sun, Mu Sun

**Affiliations:** 1 Neurodegeneration Discovery Performance Unit, GSK, R&D Shanghai, Building 1, 917 Halei Road, Zhangjiang Hi-tech Park, Pudong, Shanghai, China; 2 Department of Physiology and Pathophysiology, Shanghai Medical College, Fudan University, Shanghai, China; 3 Stem Cell Translational Research Center, Tongji Hospital, School of Medicine, Collaborative Innovation Center for Brain Science, Tongji University, Shanghai, China; University of Exeter, UNITED KINGDOM

## Abstract

NMDA receptors have been widely reported to be involved in the regulation of synaptic plasticity through effects on long-term potentiation (LTP) and long-term depression (LTD). LTP and LTD have been implicated in learning and memory processes. Besides synaptic plasticity, it is known that the phenomenon of gamma oscillations is critical in cognitive functions. Synaptic plasticity has been widely studied, however it is still not clear, to what degree synaptic plasticity regulates the oscillations of neuronal networks. Two NMDA receptor antagonists, ketamine and memantine, have been shown to regulate LTP and LTD, to promote cognitive functions, and have even been reported to bring therapeutic effects in major depression and Alzheimer’s disease respectively. These compounds allow us to investigate the putative interrelationship between network oscillations and synaptic plasticity and to learn more about the mechanisms of their therapeutic effects. In the present study, we have identified that ketamine and memantine could inhibit LTD, without impairing LTP in the CA1 region of mouse hippocampus, which may underlie the mechanism of these drugs’ therapeutic effects. Our results suggest that NMDA-induced LTD caused a marked loss in the gamma power, and pretreatment with 10 μM ketamine prevented the oscillatory loss via its inhibitory effect on LTD. Our study provides a new understanding of the role of NMDA receptors on hippocampal plasticity and oscillations.

## Introduction

The N-methyl-D-aspartate receptor (NMDAR) has long been considered to be closely linked with long-term synaptic plasticity, because of its properties of high Ca^2+^ permeability and voltage-dependent activity [1]. In the rodent hippocampus, a brain structure closely associated with processes involved in learning and memory, it is known that some forms of LTP are dependent on NMDARs, and some forms of hippocampal dependent learning and memory can be impaired by NMDAR antagonists [2]. Besides LTP, synaptic activation of NMDARs also triggers the opposite form of synaptic plasticity, long-term depression (LTD) [3] and excitotoxicity [4]. The critical role of NMDARs in synaptic plasticity, neuroprotection and excitotoxicity, has attracted extensive interest in both academia and the pharmaceutical industry to investigate the effects of NMDARs on some forms of both LTP and LTD, and their role in multiple cognition related diseases, including Alzheimer’s disease (AD).

It has been reported that several NMDAR antagonists have neuroprotective effects. One of them is memantine, which is widely prescribed for patients diagnosed with moderate-to-severe AD [5]. There is also evidence that suggests memantine has cognitive enhancing effects in other brain disorders, such as Down’s syndrome [6], Huntington’s disease [7], and autism spectrum disorder [8]. Another interesting NMDAR antagonist is ketamine which has been recently revealed to have antidepressant effect in patients and animal models [9, 10]. This novel antidepressant effect of ketamine is supported by cellular mechanisms, such as increases in synaptic transmission, spine number, synaptic proteins and BDNF expression [11, 12]. It is clear that some NMDAR antagonists have general neuroprotective effects [13], however it is still controversial whether the concentration-dependent effects of NMDAR antagonists can be explained at the level of synaptic physiology. In order to answer this question, we studied whether ketamine and memantine have a bidirectional effect on hippocampal LTP and LTD at a series of concentrations.

Besides synaptic plasticity, the phenomenon of gamma oscillations plays an important role in learning and memory function. The rhythmic electrical activities of the brain are known as oscillations and are categorized as different types according to frequency bands [14], the most ubiquitous of which are the gamma oscillations (30–90 Hz) [15]. A broad consensus is that synchronization of interneuron activity entraining rhythmic inhibition to pyramidal cells, which results in synchronous fast fluctuations of membrane potential of pyramidal cells, leading to gamma oscillations [16, 17]. It is believed that the precise timing of neuronal spiking is important for coding of information [18–20], which largely depends on the gamma oscillations [21, 22]. EEG signals, as measures of brain activity reflecting macroscopic rhythmical electrical activities, are reported to be abnormal in AD patients [23]. Notably, reduced gamma oscillations of EEG have been observed in AD patients [24, 25], and also in several AD animal models [26]. In brain slice preparation, gamma oscillations can be induced by electrical stimulation [27, 28] and by chemicals, including muscarinic [29] or kainate receptor agonists [30]. Consistent with *in vivo* findings, impaired kainate induced gamma oscillations are found in hippocampal slices of AD mouse models [31]. Since both plasticity and oscillations are closely related with cognitive function, and gamma oscillations are believed to be regulated by excitatory and inhibitory synaptic activities [16, 17], it is not surprising that there has been considerable evidence supporting the interaction between oscillations and long-term plasticity. Most studies investigated the impact of oscillations on synaptic plasticity and suggested that oscillatory status could influence synaptic plasticity [32–35], however it is still not clear how synaptic plasticity regulates the network oscillations.

To address these questions, we also investigated the effects of NMDAR antagonists on synaptic plasticity and gamma oscillations. Our results showed that at certain concentrations, both ketamine and memantine can selectively reduce LTD without affecting LTP. Surprisingly, certain concentrations of ketamine had no significant effect on the power of gamma oscillations, but could protect gamma oscillations by inhibiting hippocampal LTD.

## Materials and Methods

### 1. Animals

Male C57BL/6 mice (6–8 weeks old) were used for all experiments. All studies were conducted in accordance with the GSK Policy on the Care, Welfare and Treatment of Laboratory Animals and were reviewed and approved by the Institutional Animal Care and Use Committee (IACUC) at GSK (Protocol Number: AUP 0070). “N” refers to the number of mice used in each study whereas “n” refers to the number of slices examined in all text and figures.

### 2. Preparation of hippocampal slices

#### Slice preparation for LTP recordings

Animals were anesthetized with inhaled isoflurane then were decapitated. The brains were quickly removed and submerged in cold (~4°C) and oxygenated standard artificial cerebrospinal fluid (ACSF), which was composed of the following (in mM): 119 NaCl, 2.5 KCl, 1 NaH_2_PO_4_, 26.2 NaHCO_3_, 2.5 CaCl_2_, 1.3 MgCl_2_, 11 Glucose, 310 mOsm. Dorsal hippocampi were cut transversally into 300μm thickness sections. Slices were maintained at room temperature (24°C) in a submerged holding chamber and bubbled with carbogen gas (95% O_2_, 5% CO_2_) for at least 1 hour before recording.

#### Slice preparation for LTD recordings

Animals were anesthetized with inhaled isoflurane then were decapitated. The brains were quickly removed and submerged in cold (~4°C) and oxygenated sucrose based dissection ASCF for better slice quality, which was composed of the following (in mM): 240 sucrose, 2.5 KCl, 1.25 NaH_2_PO_4_, 24 NaHCO_3_, 0.5 CaCl_2_, 7 MgCl_2_, and 7 Glucose, 320 mOsm. Ventral hippocampi were sectioned transversally in to 300 μm thickness slices and transferred to a submerge holding chamber containing standard ACSF as above. We used both dorsal and ventral hippocampi for the pilot experiment, and chose ventral hippocampi which had more stable LTD amplitude. The difference between dorsal and ventral hippocampi on LTD may due to the distinct gene expression and function of CA1 neurons [36].

#### Slice preparation for oscillation recordings

Animals were anesthetized with intraperitoneal injection of ketamine/xylazine (100 mg/kg and10 mg/kg respectively) and then perfused intracardially with 50ml of cold (~4°C) and oxygenated modified ACSF to further improve the slice quality, which was composed of the following (in mM): 230 sucrose, 3 KCl, 1.25 NaH_2_PO_4_, 24 NaHCO_3_, 2 CaCl_2_, 2 MgCl_2_, and 10 Glucose, 320 mOsm. Horizontal slices were cut and transferred to an interface holding chamber containing standard ACSF: 119 NaCl, 2.5 KCl, 1 NaH_2_PO_4_, 26.2 NaHCO_3_, 2.5 CaCl_2_, 1.3 MgCl_2_, 11 Glucose (mM), 310 mOsm (for LTD+oscillation experiment); or 126 NaCl, 3 KCl, 1.25 NaH_2_PO_4_, 24 NaHCO_3_, 2 CaCl_2_, 2 MgCl_2_, 10 Glucose (mM) 310 mOsm (for interface oscillation experiment). Slices were maintained at room temperature with moist carbogen gas for at least 1 hour before recording.

All the chemicals for dissection and normal ACSF were obtained from Sigma-Aldrich.

### 3. Electrophysiological recordings

#### Submerged chamber recordings

Field extracellular postsynaptic potential (fEPSP) recordings were made using the Slicemaster multi-slice recording system (Scientifica UK Ltd). The system has been described in detail elsewhere [37]. Recording electrodes were pulled with a Microelectrode Puller P-97 (Sutter Ins., USA), filled with ACSF, and had resistances in the range of 2–5 MΩ. fEPSPs were recorded at *stratum radiatum* of CA1 region (150μm from cell bodies layer) and evoked by platinum or tungsten bipolar electrodes placed at the Schaffer collaterals (300 μm away from recording electrode). Testing stimulus was at 0.01667 Hz for all the experiments.

To record LTP, the chamber temperature was set at 32°C and the ACSF perfusion at 2 ml/min. Thirty minutes of baseline fEPSP were recorded with a testing stimulus intensity that elicited fEPSP amplitude at 40% of maximum. LTP was induced by theta burst stimulation (TBS) consisting of a train of ten bursts of stimuli at 5 Hz with each burst composed of four pulses at 100 Hz, with the same intensity as the test stimulus for evoking fEPSPs [38]. The train was repeated four times with a 20 s interval. LTP was monitored for 1 hour after the induction.

To record cLTD, the recording temperature was set at 30°C and ACSF perfusion at 2 ml/min. Thirty minutes of baseline fEPSP were recorded with a testing stimulus intensity that elicited fEPSP amplitude at 60% of maximum. LTD was induced by perfusion of 20 μM NMDA (Sigma-Aldrich, M3262) for 3 min [39–41]. Testing stimulus was paused during NMDA perfusion and for aother 10 min to wash-out remaining NMDA. LTD was monitored for 1 hour after the induction.

To record cLTD and oscillation sequentially, the recording temperature was set at 30°C and ACSF perfusion at 10 ml/min. Thirty minutes of baseline fEPSP were recorded with a testing stimulus intensity that elicited fEPSP amplitude at 60% of maximum. For LTD induction groups, 20 μM NMDA was applied for 5 min. Testing stimulus was suspended during NMDA perfusion and resumed after 2 min of NMDA clearance. LTD was monitored for 30 minutes then 100 nM kainate (Sigma-Aldrich, K0250) was applied to induce gamma oscillations.

All data above were acquired using Clampex 10 (Molecular Device) by 10 kHz sampling, and 1 kHz low-pass filter, 1Hz high-pass filter, 100X amplified by StAmp. Ketamine (Jiangsu Heng Rui) and Memantine Hydrochloride (Sigma-Aldrich, M9292) were applied by bath perfusion at the beginning of baseline recording.

#### Interface chamber recordings

Hippocampal slices were maintained in an interface type chamber system, the temperature was set at 31°C. ACSF perfusion was at 2.5 ml/min. Two glass electrodes synchronously recorded signals at *stratum radiatum* of CA1 and CA3 regions (150 μm away from cell bodies layer) from the same hippocampal slice by the NPI recording system (NPI electronic GmbH, Germany) for 60 sec per 5min. Low-pass and high-pass filters were respectively 300 and 0.3 Hz. Data were sampled at 10 kHz and acquired using Spike2 software (CED, UK). Baseline field potentials were recorded for 1 min, and then 100 nM kainate was added into the bath perfusion solution. After 60 minutes’ induction and recording of gamma oscillations, different concentrations of ketamine were applied into ACSF followed by at least 40min recording. For the control group, oscillations were recorded for the same time period with only ACSF perfusion [31].

### Data analysis

The slope of individual fEPSP (around 30%-70% of rising time) was analyzed by Clampfit. Power spectrum was calculated by fast Fourier Transformations (FFT) at the size of 2048 by Spike2 software package (Cambridge Electronic Design, Cambridge, UK). The total gamma power was quantified as the area under the peak in the power spectra, between 15–80 Hz. Gamma frequency oscillations in mice have been reported to be of lower frequency than those seen in rats hence a low cut-off frequency of 15 Hz was used to measure area power [31]. The coherence of oscillations was analyzed by customized script of Spike2 software and was calculated from the cross spectral density (csd) between the two waveforms (*a*, *b*) normalized by the power spectral density (psd) of each waveform. The coherence (coh) at a frequency *f* is given by:

$$\mathrm{coh}(f)=\frac{{\left|\sum csd_{ab}(f)\right|}^{2}}{\sum psd_{a}(f)\sum psd_{b}(f)}$$

Recordings with significant 50 Hz noise were excluded for this analysis. For interface oscillation experiments, the power and coherence changes were analyzed by the values 35 min after ketamine application and normalized to the values from the 10 min before ketamine application. For control groups parallel time points were analyzed according to ketamine groups. Power and coherence of oscillations were further normalized with control values. Statistical comparisons between two groups were performed by unpaired Student’s t-test using Microsoft Office Excel software; more than two groups were performed by a one way ANOVA using Sigmaplot 12.5 (Systat software). A p<0.05 was considered to indicate a statistically significant difference between groups.

## Results

### 1. Concentration-related effect of ketamine and memantine on hippocampal LTP

It is well known that NMDAR antagonists can inhibit LTP at certain concentrations. To test the concentration-related effect of ketamine and memantine on LTP, we perfused a range of concentrations of ketamine and memantine (1 μM-30 μM) on hippocampal slices. fEPSP was evoked by Schaffer Collateral stimulation and recorded at *stratum radiatum* of the CA1 region. A four-chamber Slicemaster system was used for the fEPSP recordings with successful rate of trials around 50–75%.

**Fig 1:**
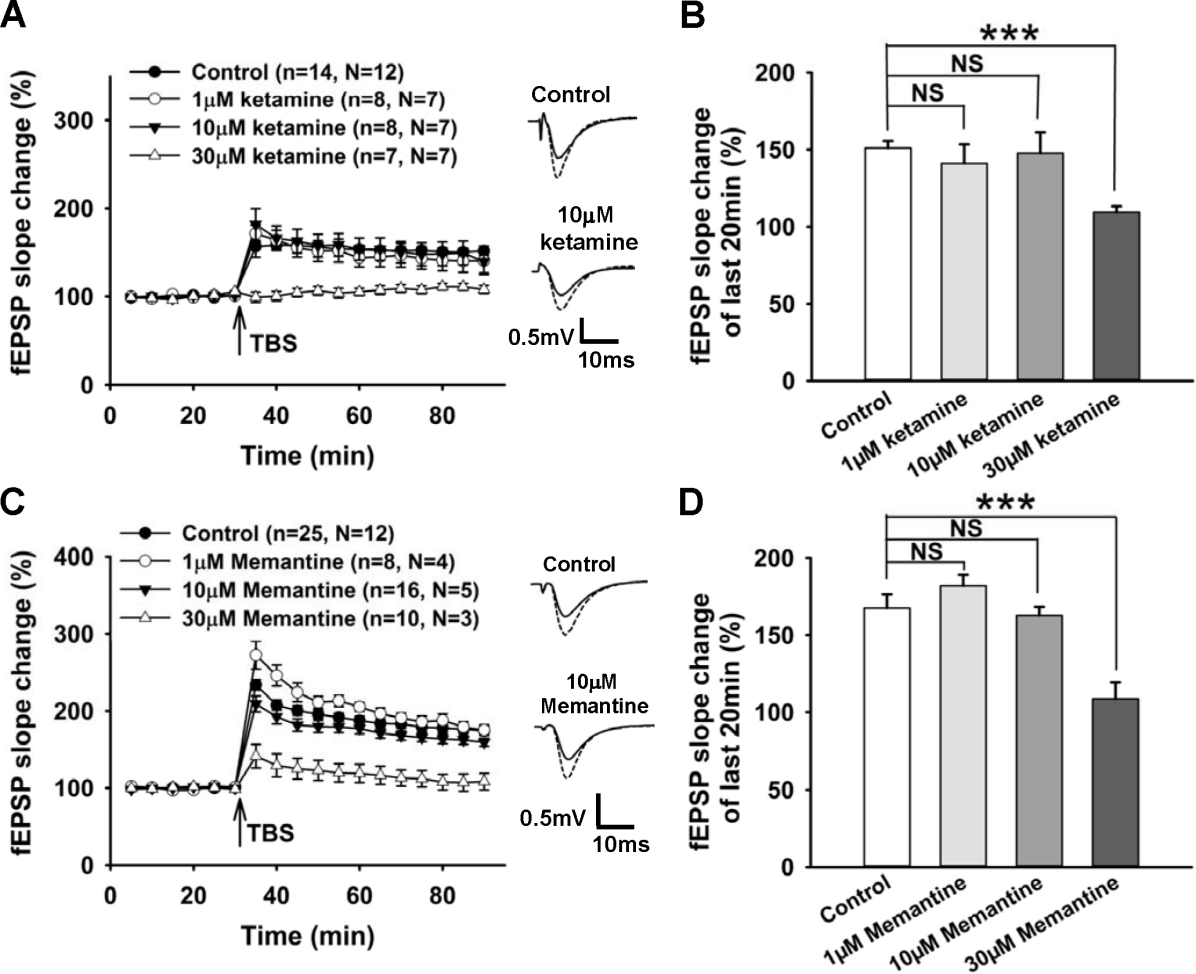
Low concentrations of ketamine and memantine did not affect LTP in the mouse hippocampal CA1 region, while higher concentrations of ketamine and memantine inhibited LTP. **A,** Effects of different concentrations of ketamine (1 μM; 10 μM; 30 μM) on LTP. Baseline of fEPSP (normalized to the average value of baseline) was recorded for 30 min followed by TBS (indicated by the arrow), and another 1 hr fEPSP was recorded after induction. Ketamine was applied before the baseline recording. **Insets,** representative fEPSP traces of 5 min before TBS (solid curve) over fEPSP traces of 55–60 min (dotted curve) after TBS for control group (upper) and 10 μM ketamine group (lower). **B,** Summarized data showing average slope change (normalized to baseline value) of the last 20 min recordings from each group. **C,** Effects of different concentrations of memantine (1 μM; 10 μM; 30 μM) on LTP. Memantine was applied before the baseline recording. **Insets,** representative fEPSP traces for control group (upper) and 10 μM memantine group (lower). **D,** Summarized data showing average slope change (normalized to baseline value) of the last 20 min recordings from each group. All data are expressed as mean ± SEM; **p<0.01, ***p<0.001, NS, no significant difference.

Hippocampal slices were treated with different concentrations of ketamine (1–30 μM), that had been reported not to disturb CA3-CA1 basal synaptic transmission [42]. The compound was bath applied before the start of fEPSP recording and within the recording period. TBS induced stable LTP in the control group, 1 μM ketamine group, and 10 μM ketamine group. Only 30 μM ketamine blocked LTP significantly (Fig 1A). Fig 1B summarizes the change of fEPSP slope during the last 20 min for each group (control 151.1 ± 4.7% of baseline, n = 14, N = 12; 1 μM ketamine 141.0 ± 12.5% n = 8, N = 7; 10 μM ketamine 147.7 ± 13.6% n = 8, N = 7; 30 μM ketamine 109.4 ± 3.9% n = 7, N = 7). The effects of memantine on CA1 LTP were also tested with the same protocol. TBS induced stable LTP in the control group as well as in the 1 μM and 10 μM memantine groups (Fig 1C). Similar to the results from the ketamine experiments, 1 μM and 10 μM memantine did not impair LTP, while a higher concentration of memantine (30 μM) significantly inhibited LTP (p<0.001). Fig 1D summarizes the fEPSP slope change of the last 20 min in each group (control 173.9 ± 5.1% of baseline, n = 25, N = 12; 1 μM memantine 182.0 ± 7.3% n = 8, N = 4; 10 μM memantine 162.7 ± 5.5% n = 16, N = 5; 30 μM memantine 108.7 ± 10.7% n = 10, N = 3).

### 2. Ketamine and memantine blocked hippocampal LTD

**Fig 2:**
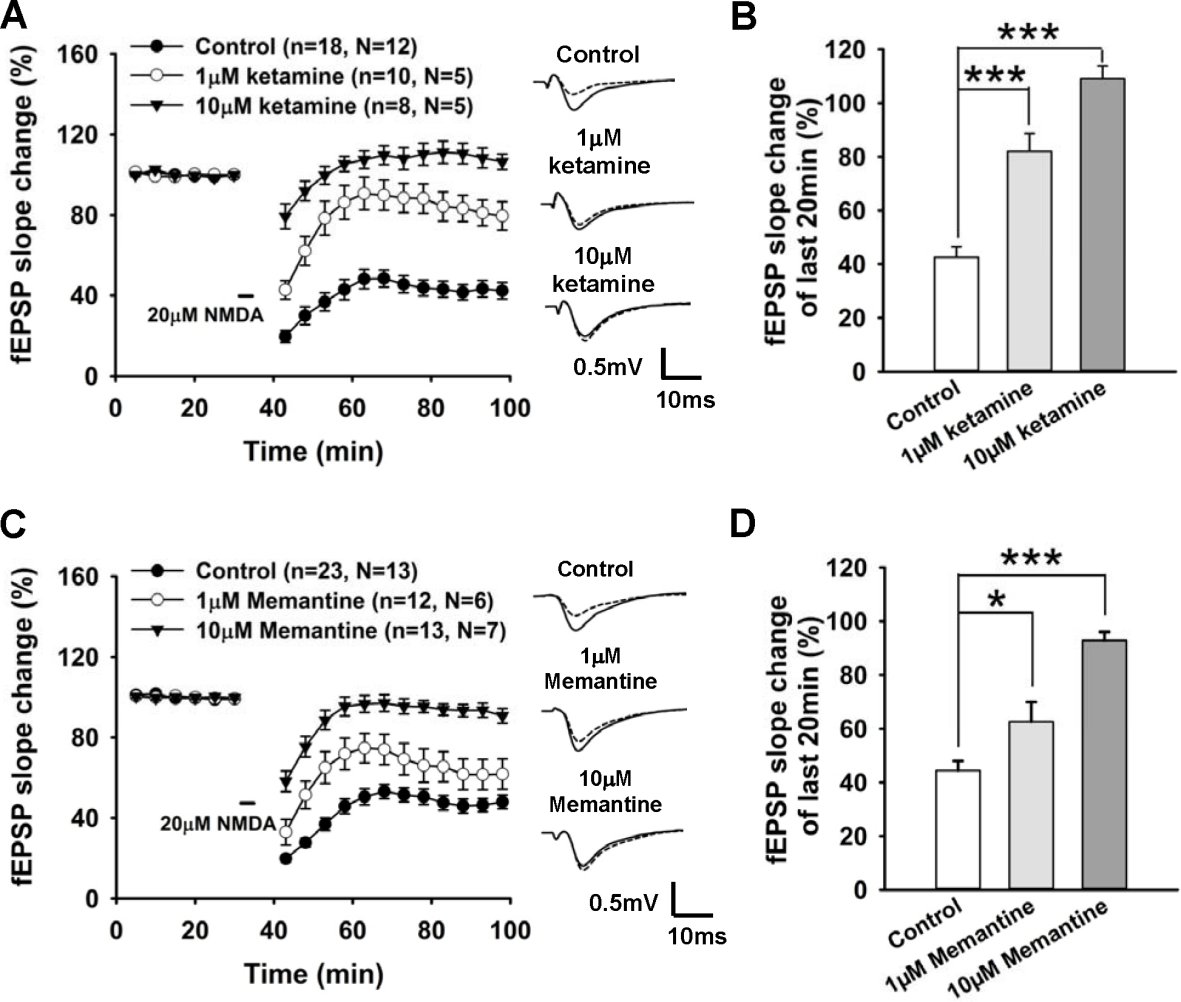
1 μM and 10 μM ketamine and memantine reduced LTD in hippocampal CA1 region. **A,** Effect of 1 μM and 10 μM ketamine on LTD. Baseline fEPSP was recorded for 30 min followed by 3 min perfusion of 20 μM NMDA (indicated by the horizontal bar). Field stimulation and recording was suspended during NMDA perfusion, resumed 10 min after NMDA application, and sustained for another 1 hr. Ketamine was applied before the baseline recording. **Insets,** representative fEPSP traces of 5 min before NMDA application (solid curve) over fEPSP traces of the last 20 min recording (dotted curve) for control group (upper), 1 μM ketamine group (middle) and 10 μM ketamine group (lower). **B,** Summarized data showing average slope change (normalized to baseline value) of the last 20 min recordings from each group. **C,** Effect of 1 μM and 10 μM memantine on LTD with the same protocol was used for ketamine. Memantine was applied before the baseline recording. **Insets,** representative fEPSP traces of 5 min before NMDA application over averaged fEPSP of the last 20 min recordings for control group (upper), 1 μM memantine group (middle) and 10 μM memantine group (lower). **D,** Summarized data showing averaged slope changes (normalized to baseline value) of the last 20 min recordings following NMDA application for control group and different memantine dose groups. All data are expressed as mean ± SEM; **p<0.01, ***p<0.001.

To investigate the effects of representative NMDAR antagonists on the counterpart of LTP, we also tested the effects of ketamine and memantine (1–10 μM) on LTD. The concentration range chosen did not inhibit LTP in our previous results. Chemical LTD (cLTD) was induced by 3 min perfusion of 20 μM NMDA to hippocampal slices. Transient NMDA perfusion induced a stable cLTD in CA1 of control group (Fig 2A). Both 1 and 10 μM ketamine significantly reduced the NMDA-induced LTD in comparison to the control group (p<0.001). The cLTD was largely reduced in the 1 μM ketamine group and totally blocked by 10 μM ketamine. Fig 2B shows the fEPSP slope change of the last 20 min in each group (control 42.5 ± 4.0% of baseline, n = 18, N = 12; 1 μM ketamine 82.0 ± 4.8% n = 10, N = 5; 10 μM ketamine 109.1 ± 6.7% n = 8, N = 5).

Memantine showed a similar effect on LTD as ketamine. As can be seen in Fig 2C, NMDA perfusion induced stable LTD in the control group, and cLTD was largely reduced in 1 μM and 10 μM memantine groups. Fig 2D shows the fEPSP slope change of last 20min in each group. Both 1 μM and 10 μM memantine significantly reduced NMDA-induced LTD in the hippocampus (control 44.4 ± 3.6% of baseline, n = 23, N = 13; 1 μM memantine 62.6 ± 7.4% n = 12, N = 6; 10 μM memantine 92.9 ± 3.1% n = 13, N = 7. p<0.05 for Control versus 1 μM group, p<0.001 for control versus 10 μM group).

Consistent with former studies [43–46] that ketamine and memantine have similar effects on synaptic plasticity, we revealed that both compounds have a concentration window (1 μM-10 μM) that selectively reduces cLTD without impairing LTP. It is likely that selectively blocking synaptic depression and maintaining normal synaptic potentiation could be beneficial for learning and memory at the synaptic level, since LTP deficits and enhancement of LTD are reported in AD pathology [26] and also in the aging process [47].

### 3. Ketamine affected CA3-CA1 coherence of gamma oscillations in mouse hippocampus

**Fig 3:**
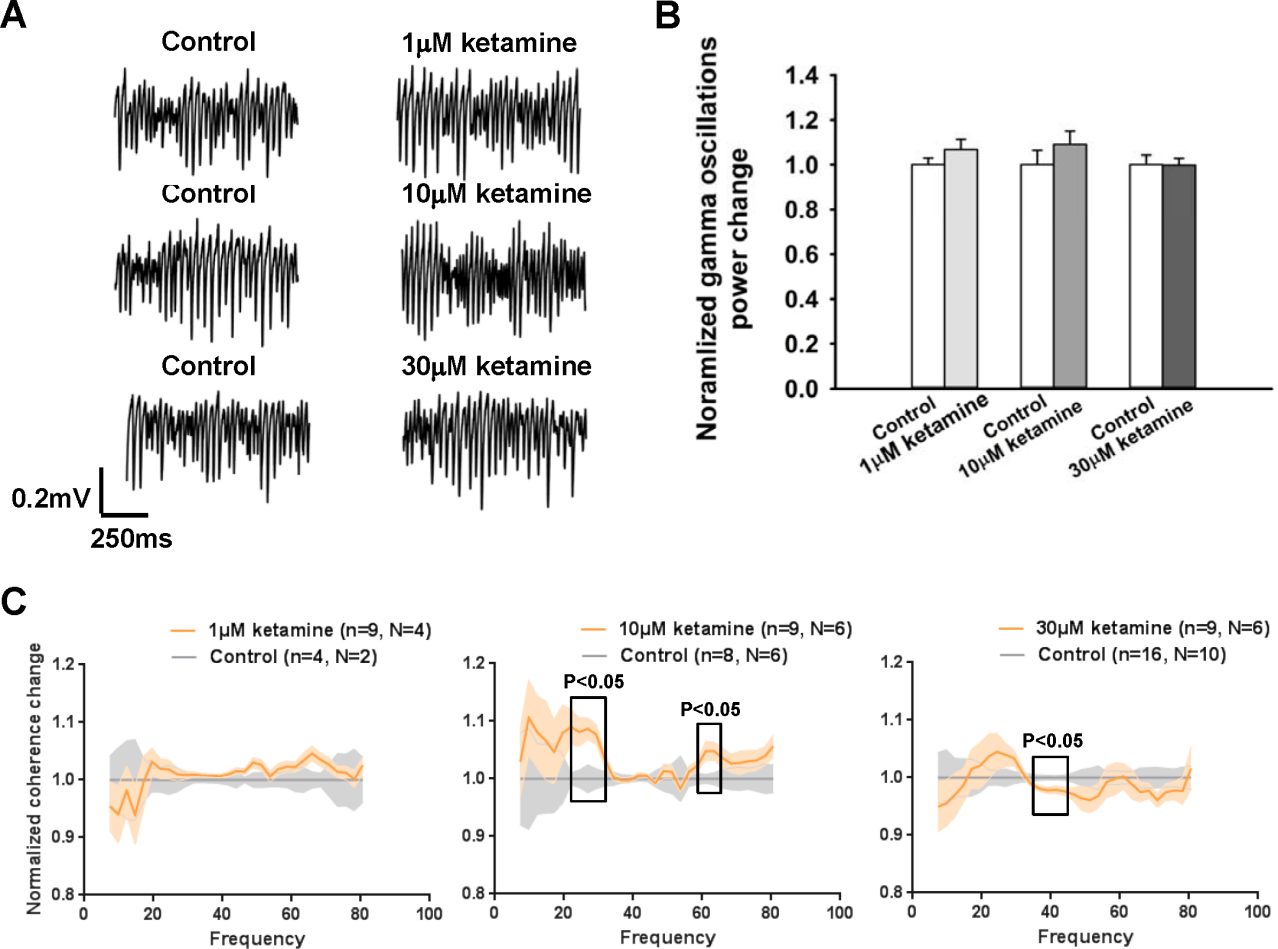
Ketamine did not affect the power of gamma oscillations but affected CA3-CA1 coherence under interface recording configuration. **A,** Representative field potential recordings in CA3 from control and different ketamine concentration groups after 90 min of 100 nM kainate treatment. Ketamine was applied 60min after kainate application. **B,** group data of normalized gamma band change in CA3 region with or without ketamine treatment. **C,** group data of normalized CA3-CA1 coherence change with or without ketamine treatment. 1 μM ketamine and its control, left; 10 μM ketamine and its control, middle; 30 μM ketamine and its control, right. All data are expressed as mean ± SEM.

Besides synaptic plasticity, such as LTP and LTD, the oscillatory activity of neuronal networks of the brain is another important electrophysiological phenomenon, which is closely related with learning and memory. Since we have shown the specific effects of ketamine and memantine on LTP and LTD, it is intriguing to know whether either of these two NMDAR antagonists can also affect gamma oscillations, especially at the concentration range of 1–10 μM. We tested the effects of different concentrations of ketamine on kainate-induced gamma oscillations in the CA3 and CA1 region of hippocampal slices in an interface recording system. In order to have better control quality, each concentration of a treatment group was compared with its own control group which was conducted on the same batch of animals. No significant difference was detected from all these groups, after 1–30 μM ketamine treatment (Fig 3A & 3B). The normalized changes of total gamma oscillation power (15–80 Hz) with or without the 35 minute ketamine perfusion were: 1 μM ketamine 1.069 ± 0.045% n = 10, N = 4 related to its control group 1 ± 0.031% n = 4, N = 2; 10 μM ketamine 1.090 ± 0.061% n = 9, N = 7 related to its control 1 ± 0.065% n = 8, N = 6; 30 μM ketamine 0.998 ± 0.032% n = 14, N = 9 related to its control 1 ± 0.043% n = 16, N = 10. There was no significant change of the peak frequencies of gamma oscillations in either CA3 or CA1 regions (data not shown). In summary, the experiments showed that 1–30 μM ketamine did not affect the power and frequency of gamma oscillations induced by kainate within 30 min in hippocampal CA3 and CA1 regions.

Since ketamine did not affect the total power of gamma oscillations, next we examined whether ketamine affected the coherence of gamma oscillations between CA3 and CA1 regions of the hippocampus. Coherence of oscillations between different regions reflects how information transfer is coordinated across several neuronal networks, and it has been reported that the coherence of CA3-CA1 oscillations is correlated with cognitive activity [48]. Fig 3C presents the normalized coherence change for the different groups. 1 μM ketamine did not affect CA3-CA1 coherence (control n = 4, N = 2; 1 μM ketamine n = 9, N = 4). However, 10 μM ketamine significantly increased coherence at 22.0–29.3Hz (22.0–26.9Hz p<0.05; 29.3Hz p<0.01) and 61–63.5Hz (p<0.05) (control n = 8, N = 6; 10 μM ketamine n = 9, N = 6). Surprisingly, 30 μM ketamine significantly reduced coherence at 36.6–43.9Hz (36.6Hz p<0.05; 39.1–41.5Hz p<0.001; 43.9Hz p<0.05) (control n = 16, N = 10; 30μM ketamine n = 9, N = 6). Although ketamine did not affect the power of gamma oscillations, 10 μM and 30 μM ketamine had significant effects on CA3-CA1 gamma coherence.

### 4. Ketamine application prevented cLTD-induced loss of gamma oscillations

Ketamine (1–30 μM) significantly modulated synaptic plasticity, however since it did not affect the total power of hippocampal gamma oscillations directly, we speculated whether ketamine could modulate gamma oscillations indirectly through its inhibitory effects on LTD.

**Fig 4:**
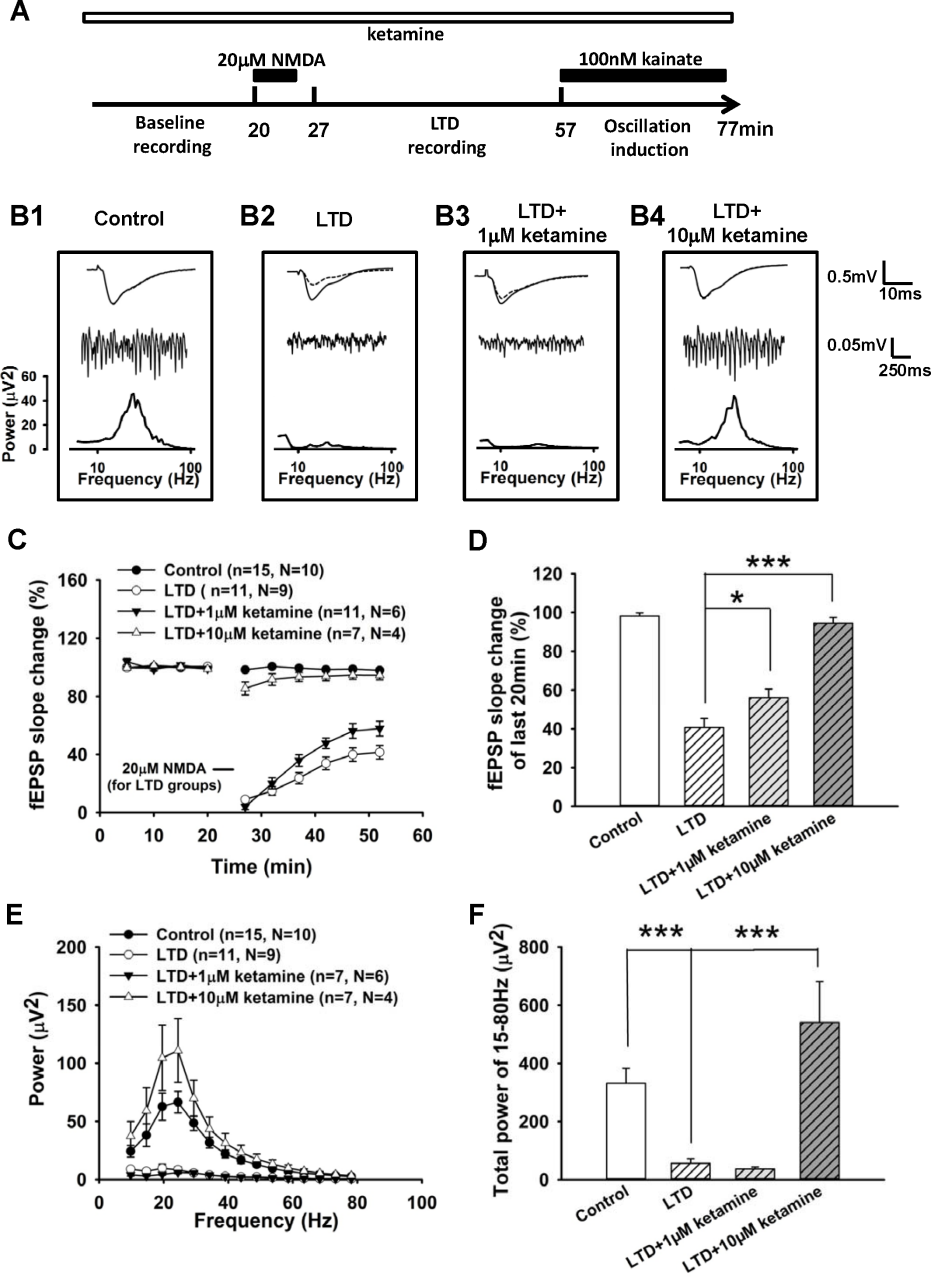
cLTD reduced hippocampal gamma oscillations and this reduction was prevented by 10 μM ketamine. **A,** The scheme is for the recording of both LTD and gamma oscillations in a hippocampal slice, ketamine was added at the beginning of the experiment (the unfilled horizontal bar). **B1, B2, B3, B4,** Representative recordings from one slice of control group (B1); LTD group (B2); LTD+1 μM ketamine group (B3); LTD+10 μM ketamine group (B4). Each column includes trace of evoked fEPSP (upper row), local field potential in CA1 (middle row) and power spectrum analysis of oscillations (lower row) from one slice respectively. Upper, representative fEPSP traces (parallel time point for control group) over averaged fEPSP of the last 20 min recording; middle, example local field potential traces showing oscillatory activity 20 min after kainate application from the same slices; lower, power spectrum of oscillations on the same slices at the same time point. **C,** Time-course of normalized fEPSP slope (normalized to baseline value) showing the control recording; LTD induction and the effects of 1 μM and 10 μM on NMDA-induced LTD. 5 min of 20 μM NMDA perfusion was indicated by the bar for LTD groups. **D,** Summarized data showing average slope change (normalized to baseline value) of the last 10 min recordings for control group, 1 μM ketamine group, 10 μM ketamine group, LTD group, LTD+1 μM ketamine group, LTD+10 μM ketamine group. **E,** Averaged power spectrum of 30 sec oscillations after 20 min of kainate application in control group, LTD group, LTD+1 μM ketamine group, LTD+10 μM ketamine group. **F,** Summarized data showing total power of 15–80 Hz oscillations in control group, LTD group, LTD+1 μM ketamine group, LTD+10 μM ketamine group. All data are expressed as mean ± SEM; *p<0.05, ***p<0.001.

In order to answer this question, we designed a new protocol to record both cLTD and gamma oscillations in the Slicemaster system since drug application efficiency is too low in the interface system to induce LTD by NMDA perfusion. We successfully induced cLTD (20 μM NMDA perfusion for 5 min) and gamma oscillations (100 nM kainate) on the same hippocampal slices sequentially. Fig 4A shows the time points of drug application and recording configurations of the LTD plus oscillation protocol. Twenty minutes after stable baseline recording, LTD was induced by 5 min perfusion of NMDA and then monitored for 30 min. Then 100 nM kainate was applied to induce gamma oscillations. For the ketamine groups, 1 μM or 10 μM ketamine was applied into bath ACSF before baseline recording and maintained through the whole experiment, including LTD phase and oscillation phase (Fig 4A). A control group was carried out with oscillations induced by kainate but the 20 μM NMDA perfusion was replaced with normal ACSF.

From slices treated with 5 min NMDA perfusion for cLTD induction, gamma oscillations were dramatically reduced. Fig 4B2 shows a representative recording of reduced fEPSP slope 30 min after LTD induction and reduced gamma oscillations compared with the recordings from a control slice (Fig 4B1). 1 μM ketamine reduced LTD significantly but did not block the loss of gamma oscillations (Fig 4B3), while 10 μM ketamine reduced LTD and thus prevented the loss of oscillations (Fig 4B4). Summarized data of fEPSP slope change from different groups are presented in Fig 4D (control 98.3 ± 1.4% baseline n = 15, N = 10; LTD 40.7 ± 4.7% baseline n = 11, N = 9; LTD+1μM ketamine 56.2 ± 4.3% baseline n = 13, N = 6; LTD+10μM ketamine 94.6 ± 3.0% baseline n = 7, N = 4). Consistent with the previous study (Fig 2A and 2B), 1 μM and 10 μM ketamine reduced cLTD in this different recording condition. Averaged power spectrums of gamma oscillations (30 sec epoch; 20 min after 100 nM kainate application) are shown in Fig 4E; total power of 15–80 Hz is shown in Fig 4F (control 331.1 ± 52.0 μV^2^ n = 15, N = 10; LTD 56.4 ± 15.7μV^2^ n = 11, N = 9; LTD+1μM ketamine 37.2 ± 6.2μV^2^ n = 7, N = 6; LTD+10μM ketamine 541.3 ± 140.4μV^2^ n = 7, N = 4). LTD markedly blocked gamma oscillations induced by kainate (p<0.0001), while 10 μM ketamine significantly protected gamma oscillations by the inhibition of LTD (p<0.0001).

There was a trend for the total gamma power of the LTD+10 μM ketamine group to be larger than that of the control group, although this did not reach statistical significance. Previous results from interface recording experiments indicated that 1 μM and 10 μM ketamine did not affect gamma power. However, it might be possible that a longer application of ketamine would have affected gamma power. In order to exclude this possibility, we further tested the effects of 1 μM and 10 μM ketamine (>1h treatment, with 0.0167Hz testing stimulation) on 100 nM kainate-induced gamma oscillations in the submerged slice chamber recording system. However longer ketamine application did not affect the power of gamma oscillations either (S1 Fig).

## Discussion

Our studies have demonstrated that two NMDAR antagonists, ketamine and memantine, within a concentration window of 1–10 μM, block LTD selectively without impairing LTP. In addition, although ketamine does not affect kainate-induced hippocampal gamma oscillations directly, it can prevent the loss of gamma oscillations through occlusion of cLTD.

We have shown that 1–10 μM ketamine and memantine inhibited LTD but did not affect LTP, while higher concentration of ketamine and memantine (30 μM) significantly reduced LTP. Consistent with several previous studies, 1–10 μM memantine did not impair LTP in rodent hippocampal slices [45, 46]. However, current result of ketamine on LTP seems inconsistent with the recent studies [42, 43], in which 10 μM ketamine significantly reduced hippocampal LTP. This discrepancy could be explained by the species difference between rats and mice, or the different protocols for LTP induction. In these two published studies [42, 43] LTP was induced by high frequency stimulation (HFS), whereas in our experiments theta burst stimulation (TBS) was used to induce LTP. The effect of ketamine on LTP/LTD in our results is consistent with that observed by Izumi and Zorumski [44], however the species and the methods of drug application were different between the two studies. We conclude that cLTD is more sensitive to the treatment of ketamine and memantine than LTP.

For ketamine, the anesthetic dose in mice is around 100 mg/kg and the corresponding maximal total serum concentration is around 30 μg/ml which can be estimated as a serum concentration around 100 μM [42, 49]. Thus, the concentrations of ketamine studied here are under the serum concentration used for anesthetization. Several studies have reported that low-doses of ketamine in mice (10 mg/kg or 3 mg/kg) have antidepressant-like effects and improve synaptic function by increasing the expression of BDNF [11, 12]. According to a previous study, corresponding maximal serum concentrations for 3–10 mg/kg ketamine is approximately 3–10 μM [49]. As a result, we chose a concentration window (1–30 μM) covering 3–10 μM for our *in vitro* study. It is interesting that this concentration range is close to our ketamine concentration window (1–10 μM) that can selectively inhibit LTD over LTP. The therapeutic concentration for memantine is also critical according to previous work, and it is known that memantine at low micro molar concentrations has beneficial effects without significant side-effects in patients [50, 51]. It is reported that therapeutically relevant doses of memantine in dementia patients yielded CSF concentration of 0.2–0.3 μM after 1–2 weeks of treatment [52]. In another study conducted in rats, a continuous treatment of memantine yielded a steady-state brain extracellular fluid concentration of approximately 0.8 μM [53], and the same treatment regimen has been reported to show cognitive benefit in AD animal models [54]. It seems that chronic exposure of a low concentration (<1 μM) of memantine has therapeutic effects. In a prior study with hippocampal slices, disruption of LTP induction by magnesium removal was restored by memantine at 1 μM, whereas LTP was further disrupted by 30 μM memantine [55]. Based on this prior result, we were interested to see the effects of 1–30 μM memantine on our LTP/LTD experiments. Several studies have reported biphasic effects of memantine on cognitive related electrophysiological and behavioral tests in rodents and the lower doses of memantine which have beneficial effects for cognition are within the concentration window that has the selectivity for LTD over LTP revealed in our study [55–57]. This indicates that the inhibitory selectivity on LTD over LTP may be related to ketamine’s and memantine’s beneficial effects on neurons and cognition. This hypothesis is further supported by the fact that LTD is enhanced and LTP is impaired in several AD animal models [26].

It is already known that acute application of ketamine can increase the power of gamma oscillations in rodents under *in vivo* conditions [58] and it has been reported that 100 μM ketamine significantly increases gamma power induced by focal application of kainate in mouse prelimbic cortex [59]. Previous studies have also shown that 10–200 μM ketamine did not affect the frequency of gamma oscillations [60–62] in hippocampal slices. Thus, we were interested to know whether 1–10 μM ketamine would affect gamma oscillations in mouse hippocampus. Our results showed that low concentrations of ketamine (1–30 μM) did not affect power and frequency of kainate-induced gamma oscillations in both CA3 and CA1 of mouse hippocampal slices, however, 10 μM and 30 μM ketamine increased and reduced CA3-CA1 gamma coherence respectively. It is postulated that one of the functions of gamma oscillations is coupling neighboring or distant cortical regions [14, 63] which is referred to as communication through coherence [64, 65]. The coupling/decoupling of brain oscillations and oscillatory coherence are related with neural communication, information transfer between different brain regions, and also involved in several cognition related diseases [65, 66]. An *in vivo* study has shown that CA3 and CA1 gamma coupling occurs during memory task performance [48]. Thus, it is interesting that we observed subtle but significant effects of ketamine on CA3-CA1 gamma coherence in our *dual* recording assay. The peak frequency of gamma oscillations in mice is lower than that in rats [31], and also largely influenced by temperature (240°C, 17.1 ± 0.4 Hz; 29°C, 23.8 ± 0.5 Hz; 34°C, 34.6 ± 0.7 Hz; 39°C, 47.7 ± 1.1 Hz) [67]. In our study the temperature for oscillation recording was 30–31°C, so the change in coherence between 22 to 29Hz represents the major part of gamma oscillations. Further studies on the coherence analysis from multiple hippocampal circuits will unveil the underlying mechanism. It is notable that only 10 μM ketamine significantly increased gamma coherence around 30 Hz, selectively reduced LTD and also prevented gamma oscillation loss after cLTD.

The novel finding of our study is that cLTD dramatically reduced hippocampal gamma oscillations and 10 μM ketamine could prevent this LTD-induced oscillatory loss. There already have been many studies focused on the relationship of oscillations and plasticity, and it is reported that the states of network oscillations regulate synaptic plasticity. We firstly observed that the loss of gamma oscillations is induced by the cLTD. Both NMDA-induced LTD and low frequency stimulation induced LTD are NMDAR dependent and widely used for synaptic physiology. Our pilot experiment showed the electrical low-frequency stimulation induced LTD was too small to differentiate drug effects, so we adopted NMDA-induced standard chemical LTD in current studies. Firstly, both synaptic plasticity and network oscillations are believed to be responsible for memory encoding at different levels, namely cellular level and network level. Our results support the association between the macroscopic phenomena of oscillations and the microscopic phenomena of synaptic plasticity. Further *in vitro* and *in vivo* studies are needed to confirm this impact of synaptic depression on network oscillations. Secondly, this result implies that abnormality of synaptic plasticity in pathological conditions or induced by drug application may be reflected at a network level, which encourages more analysis of EEG signals in AD patients [68]. Lastly, our finding that ketamine can prevent gamma loss induced by cLTD suggests that other compounds may influence the network dynamics via their effects on synaptic strength or gamma coherence, which calls for more mechanism research on NMDAR antagonists in neural networks.

## Supporting Information

S1 Fig 1 μM and 10 μM ketamine did not affect the power of gamma oscillations under submerged conditions. **A,** Example recordings from a, control group; **b,** 1 μM ketamine group; **c,** 10 μM ketamine group. Upper, example field potential traces showing oscillatory activity 20 min after kainate application; lower, power spectrum of oscillations on the same slices at the same time point. **B,** Summarized data showing total power of 15–80 Hz oscillations (30 sec oscillations after 20 min of kainate application) in control group (407.4 ± 45.0 μV^2^ n = 26, N = 17), 1 μM ketamine group (579.9 ± 121.8μV^2^ n = 11, N = 7), 10 μM ketamine group (547.8 ± 80.1μV^2^ n = 9, N = 7). No significant difference is detected among these groups.
(TIF)
