# Supplementary figures and images for: Ketamine Protects Gamma Oscillations by Inhibiting Hippocampal LTD
Source: PLoS One. 2016 Jul 28;11(7):e0159192. doi: 10.1371/journal.pone.0159192 (PMC4965035; doi:10.1371/journal.pone.0159192)

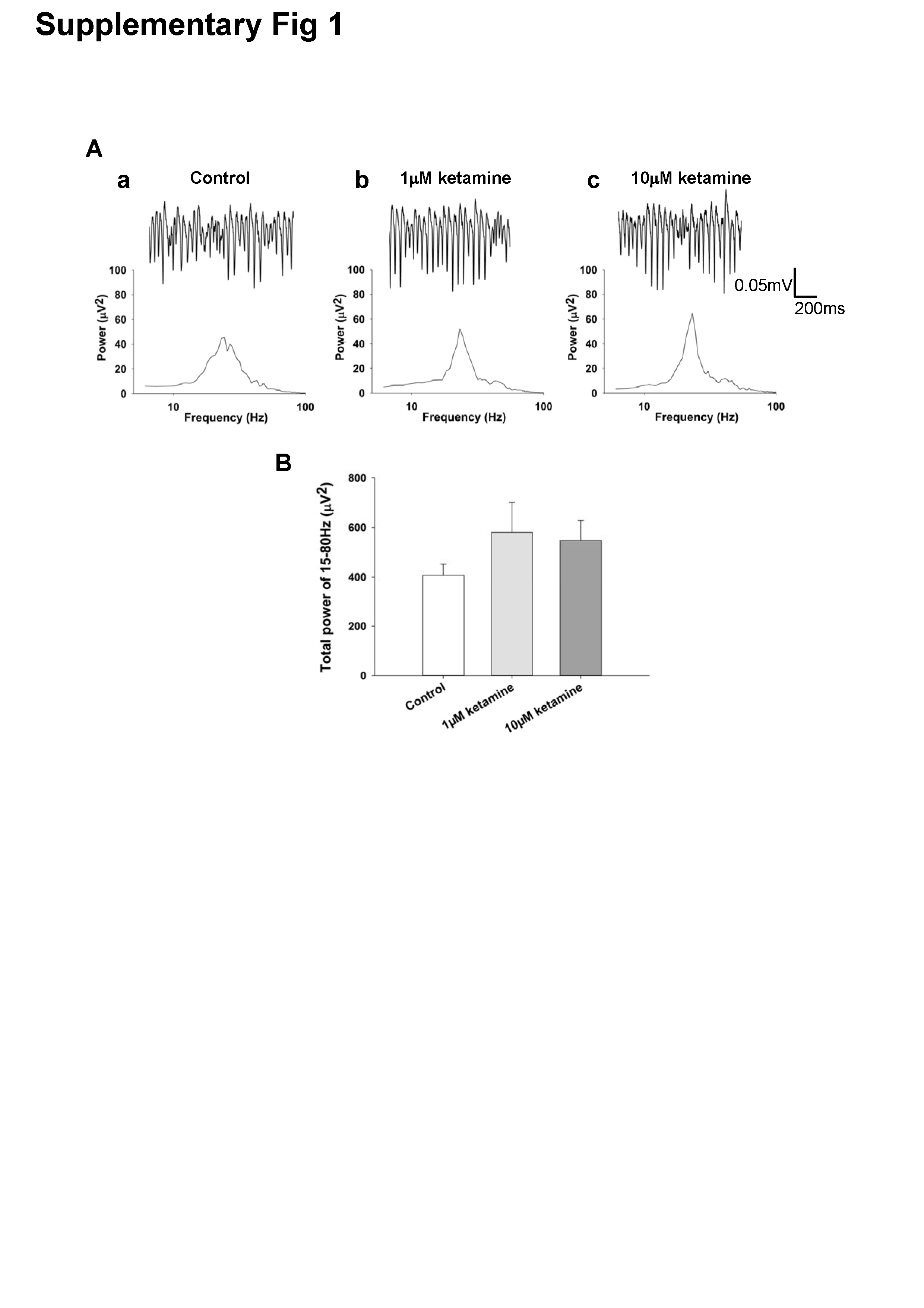

Supplement: S1 Fig — A, Example recordings from a, control group; b, 1 μM ketamine group; c, 10 μM ketamine group. Upper, example field potential traces showing oscillatory activity 20 min after kainate application; lower, power spectrum of oscillations on the same slices at the same time point. B, Summarized data showing total power of 15–80 Hz oscillations (30 sec oscillations after 20 min of kainate application) in control group (407.4 ± 45.0 μV2 n = 26, N = 17), 1 μM ketamine group (579.9 ± 121.8μV2 n = 11, N = 7), 10 μM ketamine group (547.8 ± 80.1μV2 n = 9, N = 7). No significant difference is detected among these groups. (TIF) [file pone.0159192.s001.tif]
